# Diabetische Nierenerkrankung (Update 2023): Positionspapier der Österreichischen Diabetes Gesellschaft und der Österreichischen Gesellschaft für Nephrologie

**DOI:** 10.1007/s00508-022-02147-3

**Published:** 2023-04-20

**Authors:** Harald Sourij, Roland Edlinger, Friedrich C. Prischl, Susanne Kaser, Sabine Horn, Marlies Antlanger, Bernhard Paulweber, Felix Aberer, Johanna Brix, Daniel Cejka, Harald Stingl, Alexandra Kautzky-Willer, Sabine Schmaldienst, Martin Clodi, Alexander Rosenkranz, Gert Mayer, Rainer Oberbauer, Marcus Säemann

**Affiliations:** 1 Klinische Abteilung für Endokrinologie und Diabetologie, Trials Unit für Interdisziplinäre Metabolische Medizin, Medizinische Universität Graz, Auenbruggerplatz 15, 8036 Graz, Österreich; 2 3. Medizinische Abteilung mit Stoffwechselerkrankungen und Nephrologie, Klinik Hietzing, Wien, Österreich; 3 Abteilung für Innere Medizin IV, Klinikum Wels-Grieskirchen, Wels, Österreich; 4 Universitätsklinik für Innere Medizin I, Medizinische Universität Innsbruck, Innsbruck, Österreich; 5 Abteilung für Innere Medizin, LKH Villach, Villach, Österreich; 6 Universitätsklinik für Innere Medizin 2, Kepler Universitätsklinikum Linz, Linz, Österreich; 7 Universitätsklinik für Innere Medizin I, Landeskrankenhaus Salzburg, Uniklinikum der PMU, Salzburg, Österreich; 8 Klinische Abteilung für Endokrinologie und Diabetologie, Medizinische Universität Graz, Graz, Österreich; 9 1. Medizinischen Abteilung mit Diabetologie, Endokrinologie und Nephrologie, Klinik Landstraße, Wien, Österreich; 10 Abteilung für Innere Medizin 3, Ordensklinikum Linz, Elisabethinen, Linz, Österreich; 11 Abteilung für Innere Medizin, LKH Melk, Melk, Österreich; 12 Klinische Abteilung für Endokrinologie und Stoffwechsel, Universitätsklinik für Innere Medizin III, Medizinische Universität Wien, Wien, Österreich; 13 1. Medizinische Abteilung, Klinik Favoriten, Wien, Österreich; 14 Abteilung für Innere Medizin, Krankenhaus Barmherzige Brüder Linz, Linz, Österreich; 15 Klinische Abteilung für Nephrologie, Universitätsklinik für Innere Medizin, Medizinische Universität Graz, Graz, Österreich; 16 Nephrologie und Hypertensiologie, Universitätsklinik für Innere Medizin IV, Medizinische Universität Innsbruck, Innsbruck, Österreich; 17 Klinische Abteilung für Nephrologie und Dialyse, Universitätsklinik für Innere Medizin III, Medizinische Universität Wien, Wien, Österreich; 18 6. Medizinische Abteilung mit Nephrologie & Dialyse, Klinik Ottakring, Wien, Österreich

**Keywords:** Diabetes mellitus Typ 1, Diabetes mellitus Typ 2, Diabetische Nierenerkrankung, Chronische Nierenerkrankung, Dialyse, Type 1 diabetes, Type 2 diabetes, Diabetic kidney disease, Chronic kidney disease, Dialysis

## Abstract

Epidemiologische Untersuchungen zeigen, dass etwa 2–3 % aller Österreicher*innen einen Diabetes mellitus mit Nierenbeteiligung aufweisen. Dies betrifft somit in Österreich etwa 250.000 Menschen. Das Risiko des Auftretens und Fortschreitens der diabetischen Nierenerkrankung kann durch Lebensstilinterventionen und Optimierung des arteriellen Blutdrucks, Blutzuckers und spezielle Medikamentenklassen vermindert werden. In diesem gemeinsamen Artikel der Österreichischen Gesellschaften für Nephrologie und Diabetologie werden die entsprechende Diagnostik und therapeutische Strategien bei diabetischer Nierenerkrankung vorgeschlagen.

## Abstract (en)

Epidemiological investigations have shown that approximately 2–3% of all Austrians have diabetes mellitus with renal involvement, leaving 250,000 people in Austria affected. The risk of occurrence and progression of this disease can be attenuated by lifestyle interventions as well as optimization of blood pressure, blood glucose control and special drug classes. The present article represents the joint recommendations of the Austrian Diabetes Association and the Austrian Society of Nephrology for the diagnostic and treatment strategies of diabetic kidney disease.

Die diabetische Nierenerkrankung wird anhand einer persistierenden Erhöhung der Harn-Albuminausscheidung (Albumin-Kreatinin-Ratio ≥ 30 mg/g) und/oder einer reduzierten eGFR (geschätze glomeruläre Filtrationsrate; < 60 ml/min/1,73 m^2^) bei vorliegendem Diabetes mellitus und dem Fehlen von Zeichen und Symptomen einer anderen primären Ursache für eine Nierenerkrankung diagnostiziert.

Diabetes mellitus und vaskulär-hypertensive Erkrankungen stellen die häufigsten Ursachen terminalen Nierenversagens in Österreich dar [1]. Strategien zur Verhinderung des Auftretens bzw. der Progression sind daher von größter Bedeutung. Im Jahr 2019 waren laut österreichischem Dialyse- und Transplantationsregister (OEDTR) 25,3 % der Neuzugänge zur Dialyse Menschen mit Diabetes mellitus (23,1 % Typ-2-Diabetes [T2D], 2,2 % Typ-1-Diabetes [T1D]). Es muss darauf hingewiesen werden, dass die Inzidenz der Dialysepatient*innen mit T2D seit 2007 kontinuierlich rückläufig ist, die Prävalenz jedoch weiterhin steigt oder stabil bleibt [2]. Letzteres wird durch Daten aus dem OEDTR unterstrichen, die eine Steigerung des Überlebens von T2D-Patienten im Zeitraum von 1998 auf 2007 um insgesamt ein Jahr zeigen konnten [3].

## Die Nierenerkrankung bei Patient*innen mit Typ‑1-Diabetes (T1D)

Der Verlauf der Nierenerkrankung bei Patient*innen mit T1D ist weniger variabel als bei Patient*innen mit T2D und eine optimale/intensivierte Blutzuckereinstellung ist hier die wichtigste Maßnahme zur Prävention und in frühen Stadien auch der Intervention. Bei optimaler Einstellung (HbA_1c_ < 7 % (53 mmol/mol)) kam es in einer großen Interventionsstudie nach 30 Jahren zu einer 36–76%igen Reduktion der mikrovaskulären Komplikationen im Vergleich zur Gruppe mit einem HbA_1c_ ~ 9 % [4]. Die Inzidenz der terminalen Niereninsuffizienz in der intensiv behandelten Gruppe lag bei 11/1000 Patienten [5]. Sobald entweder eine Hypertonie oder eine Albuminurie (ab Stadium A2) vorliegen, gilt die medikamentöse Blockade des Renin-Angiotensin-Aldosteron-Systems (RAAS) als gesicherte Therapie zur Nephroprotektion (Reduktion der Albuminurie und Reduktion des GFR-Abfalls) [6–9].

## Die Nierenerkrankung bei Patient*innen mit Typ‑2-Diabetes (T2D)

Die Prävalenz des T2D in Österreich ist nicht genau bekannt, liegt aber etwa bei 8 % der erwachsenen Bevölkerung. Etwa 25 % dieser Patienten haben auch eine chronische Niereninsuffizienz (CKD = „chronic kidney disease“) Stadium G3 oder höher (eGFR < 60 ml/min/1,73m^2^) [10], diese werden in weiterer Folge unter dem Begriff diabetische Nierenerkrankung (DKD = „diabetic kidney disease“) zusammengefasst. Rezente amerikanische Daten gehen davon aus, dass ca. 24 % aller Fälle von CKD (d. h. eGFR < 60 ml/min/1,73m^2^ oder Albumin/Kreatinin-Ratio ≥ 30 mg/g oder beides) nach Korrektur für demografische Faktoren durch Diabetes mellitus verursacht werden [11]. Durch das erhöhte Mortalitätsrisiko von T2D-Patient*innen („competing risk of death“) versterben viele, bevor sie das Stadium der terminalen Niereninsuffizienz erreichen.

Eine CKD bei T2D ist ätiologisch heterogener als bei T1D-Patient*innen, somit sind der Verlauf und die Prognose schwieriger abzuschätzen. Aufgrund der meist schon längeren Zeitspanne zwischen Beginn der gestörten Stoffwechsellage und Diagnose des T2D kann zum Zeitpunkt der Diagnosestellung bereits eine Albuminurie vorliegen. Ohne spezielle Intervention entwickeln ca. 20–40 % der Patient*innen eine Albuminurie Stadium A2 (s. unten) sowie eine größere Albuminurie bzw. Proteinurie (Stadium A3), die DKD schreitet aber insgesamt nur bei etwa 20 % dieser Patienten innerhalb von 20 Jahren zu einer terminalen Niereninsuffizienz fort [12]. Das Auftreten einer Albuminurie *per se* sowie das Vorliegen einer CKD gehen mit einer erhöhten Inzidenz kardiovaskulärer Morbidität und Mortalität einher [13]. Früher ging man von einem klassischen „Durchlaufen“ aller Stadien bis zur Entwicklung der terminalen Niereninsuffizienz aus und betonte die Wertigkeit der Albuminurie im Stadium A2 als Parameter der Frühdiagnostik. Bei vielen diabetischen Patient*innen mit eingeschränkten Nierenfunktionsparametern findet sich jedoch keine Albuminurie [12], sodass hier primär eine mikro-/makrovaskuläre Komponente in der Niere anzunehmen ist. Zudem werden auch unterschiedliche Albuminurie-Verläufe bis hin zu einer Regression der Albuminurie ohne spezifische Therapie bei Patienten mit Diabetes beobachtet.

## Geschichte und Spektrum der diabetischen Nierenerkrankung

Mitte des 20. Jahrhunderts wurde der Begriff der diabetischen Nephropathie als klinisches Syndrom, basierend auf interkapillärer oder nodulärer Glomerulosklerose (Kimmelstiel-Wilson) bei Patienten mit längerer Diabetesdauer, persistierender Albuminurie, Hypertonie, Retinopathie und progressivem Nierenfunktionsverlust geprägt [14, 15]. Dieser wurde in den letzten Jahren durch die klassischen fünf Stadien des natürlichen Krankheitsverlaufes einer CKD ergänzt [16]. Obwohl dieses Modell und der Krankheitsverlauf primär auf Daten von Patienten mit T1D basierte [17, 18], wurde es auch auf Patienten mit T2D angewandt [19]. Mittlerweile ist aber klar, dass mehr als 50 % der Patient*innen mit T2D in Langzeitbeobachtungen eine GFR < 60 ml/min/1,73 m^2^ ohne vorangehende Albuminurie entwickeln [20–23] bzw. der Verlauf der Albuminurie nicht immer mit dem Nierenfunktionsverlust korreliert [24]. Ähnliche Beobachtungen gibt es auch für T1D [25]. Patho-histologische Untersuchungen bei Patienten mit T2D und CKD weisen auf ein vielfältiges Spektrum an Nierenerkrankungen hin: so liegt der der Anteil einer histomorphologischen diabetischen Nephropathie bei ca. 30–50 %, dazu existieren bei ca. 20–30 % andere Nierenrkrankungen (z. B. IgA-Nephropathie, fokale segmentale Glomerulosklerose (FSGS) etc.) und schließlich findet sich noch einmal ein gemischter Anteil an eigentlicher diabetischer Nephropathie und anderen Nierenerkrankungen von ca. 20–30 %. Dabei weist das Ausmaß der Albuminurie die engste Korrelation mit dem Auftreten einer pathohistologischen diabetischen Nephropathie auf [26]. Von der *Renal Pathology Society* wurde zwar eine Klassifikation auf Basis von Biopsien von Patient*innen mit T1D und T2D erstellt [27], allerdings wird diese Klassifikation in der klinischen Routinebefundung im Allgemeinen wie auch in Österreich speziell nicht verwendet.

**Figure 1:**
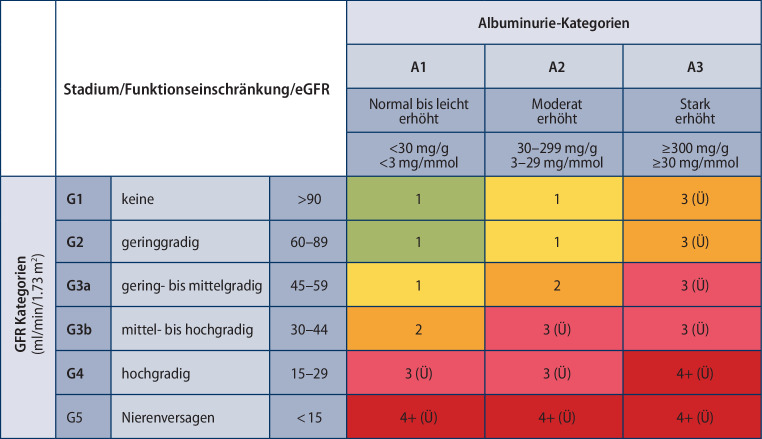

Die Stadieneinteilung der DKD entspricht der klassischen Einteilung der CKD Stadien nach KDIGO: die geschätzte glomeruläre Filtrationsrate (eGFR) wird in die Stadien G1–G5 (Abb. 1) eingeteilt, Stadium G3 in G3a (eGFR 45–59 ml/min/1,73 m^2^) und G3b (eGFR 30–44 ml/min/1,73 m^2^) unterteilt und zusätzlich wird die Albuminausscheidung im Spontanharn (Albumin/Kreatinin Ratio) in A1 (< 30 mg/g Kreatinin), A2 (30–300 mg/g) und A3 (> 300 mg/g) unterschieden. Zudem wird in der neuen Klassifizierung auch farblich das Risiko für das Auftreten kardiovaskulärer Ereignisse und das Fortschreiten der Funktionseinschränkung der Nieren bis hin zum Nierenversagen dargestellt (Abb. 1).

## Bestimmung der Nierenfunktion

Zur Beurteilung des Ausmaßes der Nierenfunktionseinschränkung sollte eine der derzeit gängigen Schätzformeln verwendet werden, welche bereits in den meisten Labors implementiert sind. Eine ausschließliche Serum-Kreatininbestimmung ist v. a. bei älteren Menschen oft irreführend, da keine gute Korrelation zur tatsächlichen Nierenfunktion besteht bzw. die Schwelle zu einer definierten Nierenerkankung vermutlich niedriger anzusetzen ist als bei jungen Menschen (45 vs. 60 ml/min/1,73 m^2^). Die mittels MDRD (Modification of Diet in Renal Disease)-Formel geschätzte glomeruläre Filtrationsrate (eGFR) ist für den Bereich zwischen 20 und 60 ml/min/1,73 m^2^ für Personen über 18 Jahren validiert [29]. Die Basis der Berechnung soll eine nach IDMS („isotope dilution mass spectrometry“)-Goldstandard kalibrierte Serum-Kreatininbestimmung sein ([30]; Tab. 1). Aktuell empfehlen die meisten Gesellschaften die CKD-EPI (Chronic Kidney Disease Epidemiology Collaboration)-Formel als Standard ([31, 32]; Tab. 1). Für diese Formel wurde mehrfach gezeigt, dass sie v. a. im CKD Stadium G2–3 genauer als die MDRD-Formel und somit besser zur Risikostratifizierung geeignet ist [33, 34].

**Table 1:** 

*Glomeruläre Filtrationsrate berechnet (eGFR) – MDRD4-Formel*
GFR (ml/min) / 1,73 m^2^ KÖF = 186 × (s_Cr_)^−1,154^ × Alter − 0,203 × (0,724 bei Frauen)
*Glomeruläre Filtrationsrate berechnet (eGFR) – CKD-EPI-Formel*
GFR = 142 × min (s_cr_ / κ, 1)^α^ × max (s_cr_ / κ, 1)^−1,20^ × 0,9938^Alter^ × 1,012 (Frauen)

*KÖF* Körperoberfläche, *s*_*Cr*_ Serumkreatinin, Frauen: κ = 0,7; α = −0,329; Männer: κ = 0,9; α = −0,411

Aufgrund der Ressourcen und Praktikabilität sind in der täglichen Praxis andere Schätzformeln z. B. unter Einbeziehung von Cystatin C derzeit von geringerer Bedeutung. Zur besseren allgemeinen Verständlichkeit schlagen die Gesellschaften vor, gegenüber Betroffenen die Nierenfunktion als % Nierenfunktion zu interpretieren, was bei einem annähernden Normalwert von etwa 100 ml/min/1,73 m^2^ (90–120 ml/min/1,73 m^2^) durchaus gerechtfertigt erscheint.

## Diagnostik der diabetischen Nierenerkrankung

### Screening auf diabetische Nierenerkrankung

Bei T1D sollte das jährliche Screening auf Albuminurie fünf Jahre nach Diagnosestellung, bei T2D bereits mit der Diagnosestellung beginnen. Generell wird empfohlen, als Screening nur die Messung der Albumin/Kreatinin-Ratio aus dem Spontanharn durchzuführen [35]. Wir empfehlen, unabhängig von der Bestimmung der Albuminurie auch eine regelmäßige eGFR-Bestimmung insbesondere bei T1D nach zumindest fünf Jahren Krankheitsdauer und bei allen T2D-Patient*innen schon ab Diagnosbeginn durchzuführen [36]. Die Harnalbumin-Ausscheidung wird mit den Stadien A1–A3 klassifiziert (siehe Abb. 1).

Aufgrund der hohen individuellen Variabilität der Albuminexkretion wird zur Diagnostik der Albuminurie folgendes Vorgehen empfohlen, dabei gilt die „2 aus 3 Regel“: Wenn zwei hintereinander analysierte Urinproben innerhalb von 3–6 Monaten übereinstimmend positiv (Albuminausscheidung > 30 mg/g Kreatinin) oder negativ sind, ist eine Albuminurie nachgewiesen bzw. ausgeschlossen. Wenn die Albuminausscheidung einer Urinprobe negativ und die andere positiv ist, sollte eine dritte Urinprobe auf Albuminurie getestet werden. Zu beachten ist, dass positive Befunde z. B. auch bei akut fieberhaften Erkrankungen, Harnwegsinfekten und arterieller Hypertonie, bei Herzinsuffizienz, Menstruation und nach körperlicher Anstrengung unabhängig von Nierenschäden möglich sind. Nachdem die Sammlung von 24-h Harn aufwendig ist und nur wenig Zusatznutzen im Vergleich zur Bestimmung der Albumin-Kreatinin-Ratio aus dem Spontanharn aufweist, hat sich letztere Methode durchgesetzt.

## Differenzialdiagnosen bei Patient*innen mit diabetischer Nierenerkrankung

Bei Menschen mit Diabetes mellitus sollte immer auch an eine mögliche andere, nicht-diabetische Ursache der Proteinurie und/oder Nierenfunktionseinschränkung gedacht werden, insbesondere wenn mindestens eines der folgenden Kriterien erfüllt ist:

- Diabetesdauer unter 5 Jahren bei T1D,
- fehlende (insbesondere proliferative) oder nur milde diabetische Retinopathie,
- pathologisches Harnsediment mit Mikrohämaturie (insbesondere Akanthozytennachweis und Erythrozytenzylinder),
- sehr rasche Zunahme der Albuminurie, definiert als Klassenwechsel der Albuminurie (A1 auf A2 oder A3 sowie A2 auf A3 innerhalb kurzer Zeit),
- rascher Anstieg des Serum-Kreatinins,
- Auffälligkeiten in der Nierensonographie, welche an eine andere Nierenpathologie denken lassen.

Differenzialdiagnostisch häufig zu erwägende Nierenerkrankungen, die statt oder auch zusätzlich zu einer DKD bestehen können, sind eine hypertensive oder eine ischämische Nephropathie als Folge einer Atherosklerose/Aterioloklerose der Nierengefäße. Bei ausgeprägter Albuminurie und/oder Mikrohämaturie ist differentialdiagnostisch an andere renale Erkrankungen zu denken (u. a. Vaskulitis, Glomerulonephritis, Amyloidose), die einer gezielten Therapie bedürfen, weshalb hier eine weitere nephrologische Abklärung notwendig ist. Eine Indikation zur Nierenbiopsie als diagnostischer Goldstandard sollte in Anbetracht der Vielfalt anderer ernsthafter Nierenerkrankungen in diesen Fällen großzügig gestellt werden.

## Therapeutische Gesichtspunkte bei Patient*innen mit diabetischer Nierenerkrankung

### Ernährung

Hinsichtlich der Eiweißzufuhr mit der Nahrung werden nach den KDIGO- und den ADA-Leitlinien 0,8 g/kg Körpergewicht sowie die Vermeidung der Überschreitung von 1,3 g/kg Körpergewicht empfohlen [37]. Eine noch niedrigere Eiweißzufuhr scheint keinen weiteren Nutzen zu haben [38]. Zusätzlich wird ein Meiden von industriell verarbeiteten Lebensmitteln [39] empfohlen sowie eine Reduktion der Kochsalzzufuhr auf 5 g/d vorgeschlagen [37]. Es sollte dazu angemerkt werden, dass auch Studien bei T1D- und bei T2D-Patient*innen die Kochsalzrestriktion kritisch hinterfragen, und die Studienlage in dieser Population dafür nur geringe Evidenz liefert [37, 40, 41]. Weitere Studien zum therapeutischen Nutzen einer Kochsalz-Restriktion sind daher notwendig, bevor solide Empfehlungen gemacht werden können.

Verschiedene Diäten werden diskutiert, um das kardiovaskuläre Risiko der Patient*innen mit Diabetes mellitus zu senken. Diäten sind naturgemäß schwierig zu standardisieren und die Studienlage ist heterogen. Eine allgemeingültige Diätempfehlung, die für jede Patient*in gut passend und auch praktisch umsetzbar ist, kann aktuell nicht gegeben werden [42–45]. Gesichert scheint aber ein allgemein gesunderer Lebensstil zur Verhinderung bzw. Progressionsverzögerung der Nierenerkrankungen bei Personen mit Diabetes mellitus zu sein [46].

Gewichtsreduktion bei morbider Adipositas (BMI > 40 kg/m^2^) durch ein bariatrisch chirurgisches Vorgehen [47] führt zu einer Verbesserung der Stoffwechsellage oder sogar zur Diabetesremission und zu einer deutlichen Reduktion der Risken für diabetische Komorbiditäten [48].

### Kardiovaskuläres Risiko

Bei bestehendem Diabetes mellitus ist bei Nierenerkrankung konsistent eine substanzielle Erhöhung der Mortalität beobachtet worden [13]. Ein Großteil der erhöhten Mortalität ist auf kardiovaskuläre Erkrankungen zurückzuführen, obwohl die nicht-kardiovaskuläre Mortalität ebenso erhöht ist. Albuminurie und eGFR sind unabhängige und zusätzlich assoziierte Risikofaktoren für kardiovaskuläre Ereignisse, kardiovaskuläre Mortalität und Gesamtmortalität [13, 49]. Sowohl Diabetes mellitus als auch CKD bewirken vergleichbare Inzidenzraten von kardiovaskulären Ereignissen wie Patient*innen mit manifester koronarer Herzerkrankung [50]. Dies führt zur Empfehlung, dass Patient*innen mit Diabetes mellitus, CKD oder diabetischer Nierenerkrankung präventiv hinsichtlich kardiovaskulärer Erkrankungen so behandelt werden sollen, als ob sie bereits ein solches Ereignis erlitten hätten [50]. Diese Beobachtungen ziehen nach sich, dass Behandlungsstrategien darauf ausgerichtet sein sollen, das hohe kardiovaskuläre Risiko von Patient*innen mit DKD abzuschwächen, um letztlich das Überleben zu verbessern [51]. Die Mechanismen, durch die eine DKD das kardiovaskuläre Risiko beeinflusst, umfassen traditionelle Risikofaktoren (Hyperglykämie, Hypervolämie und Hypertonie, Lipoprotein-Metabolismus, systemische Inflammation, oxidativer Stress und endotheliale Dysfunktion) wie auch Mechanismen spezifisch im Zusammenhang mit der Nierenfunktionseinschränkung (z. B. Urämietoxine, Anämie und Störungen des Knochen- und Mineralstoffwechsels) [51]. Diese Überlegungen fließen in die unten angeführten Therapieempfehlungen ein.

### Lipidstoffwechsel

Die DKD wird durch Störungen des Lipidmetabolismus in Zusammenhang mit einer Abnahme der Nierenfunktion abhängig vom Stadium der CKD begleitet. Wie bereits erwähnt führt das Vorliegen einer CKD auch zu einem deutlichen Anstieg des kardiovaskulären Risikos [49]. LDL-Cholesterin ist ein etablierter Risikofaktor für kardiovaskuläre Erkrankungen in der Allgemeinbevölkerung. Sein prognostischer Wert ist allerdings bei Personen mit eingeschränkter Nierenfunktion aufgrund einer DKD eingeschränkt [52] und es wird eine quantitative Verschiebung im Lipidprofil zu erhöhten Triglyzeriden, niedrigem HDL-Cholesterin – inkl. qualitativen Änderungen des HDL Cholesterins – sowie erhöhten Spiegeln an oxidiertem LDL-Cholesterin gefunden [53].

Das Ausmaß der LDL-Senkung bei der CKD-Population mit Statinen ist vergleichbar mit Personen mit erhaltener Nierenfunktion [54]. Klinische Untersuchungen und entsprechende Meta-Analysen bei nicht-dialysepflichtiger CKD zeigen, dass kardiovaskuläre Ereignisse und Mortalität durch Statine bzw. die Kombination Statine/Ezetimibe im Vergleich zu Plazebo gesenkt werden [54, 55]. Der günstige Effekt scheint nicht durch Diabetes modifiziert zu sein. Während also der kardiovaskuläre Benefit durch Statine bei CKD gut dokumentiert ist, haben Statine keine progressionsverzögernde Wirkung hinsichtlich der Nierenfunktion [56]. Basierend auf den rezenten KDIGO-Leitlinien, werden Statine bei allen diabetischen Patient*innen mit nicht-dialysepflichtiger CKD empfohlen [57]. Rezente Daten haben auch gezeigt, dass PCSK9-Hemmer bei Personen im CKD-Stadium 3–5 (mit und ohne T2D) vergleichbare lipidsenkende Wirkungen und Sicherheitsprofile wie bei Patient*innen mit einer eGFR > 60 ml/min/1,73 m^2^ aufweisen [58] und sind daher für die Therapie in diesem Kollektiv auch zugelassen, ebenso wie die small interfering RNA-Therapie mit Inclisiran (Fachinformation 2022).

Bei Dialysepflichtigkeit haben zwei größere klinische Studien (4D und Aurora) keinen Vorteil einer Statintherapie auf den kombinierten kardiovaskulären Endpunkt (3-Punkt MACE) zeigen können [59, 60]. Im Kollektiv von Menschen mit Diabetes und Dialysepflicht zeigt sich jedenfalls keine zwingende Indikation für die Neueinleitung einer Statintherapie.

### Betreuung der Patienten mit diabetischer Nierenerkrankung

Eine nephrologische Begutachtung ist bei Unklarheit über die Ätiologie der Nierenerkrankung und/oder rascher CKD Progression indiziert. Prinzipiell sollte auch unabhängig vom CKD Stadium bei A3 sowie bei CKD G3b (zumindest ab A2) und G4 unabhängig von der Albuminurie eine nephrologische Vorstellung bzw. Betreuung erfolgen. Ab CKD Stadium G3 sollte eine gemeinsame Betreuung durch Diabetolog*innen und Nephrolog*innen erwogen werden und zusätzlich Augenmerk auf mögliche renale Folgeerkrankungen gelegt werden.

Ab CKD Stadium G4 ist auch die Eignung für eine alleinige Nierentransplantation oder eine kombinierte Nieren- und Pankreastransplantation (bevorzugt bei T1D, aber auch in ausgewählten Fällen bei T2D möglich [61]) zu prüfen. Optimal ist eine präemptive Transplantation (Lebend- oder Post-mortem-Spende), insbesondere bei Patient*innen mit T1D ist aufgrund des exzessiven kardiovaskulären Risikos eine rechtzeitige Evaluation für eine Transplantation anzustreben, um die Zeit an der Hämo- oder Peritonealdialyse so kurz als möglich zu halten.

## Antihyperglykämische Therapie (Abb. 2)

Bei Menschen mit T1D oder T2D sollte möglichst eine normoglykämische Stoffwechselsituation angestrebt werden [62]. In der Primärprävention sind niedrigere HbA_1c_-Werte zu fordern als in fortgeschrittenen Stadien der CKD und in der Sekundärprävention. Hier hat sich in den Studien ein HbA_1c_-„Zielkorridor“ von 6,5–7,5 % als sinnvoll erwiesen. Unabhängig davon sollte aufgrund der Vorgeschichte, Komorbiditäten, Hypoglykämieneigung und diabetischer Begleiterkrankungen (Retinopathie, Neuropathie) insbesondere bei älteren Patient*innen eine individualisierte Festlegung der Therapieziele erfolgen. Bei nachlassender Nierenfunktion ist besonders das erhöhte Risiko der Hypoglykämie zu berücksichtigen. Die Wahl antidiabetischer und anderer Medikamente bedarf bei eingeschränkter Nierenfunktion erhöhter Aufmerksamkeit, da Zulassungseinschränkungen und Kontraindikationen vorliegen können.

**Figure 2:**
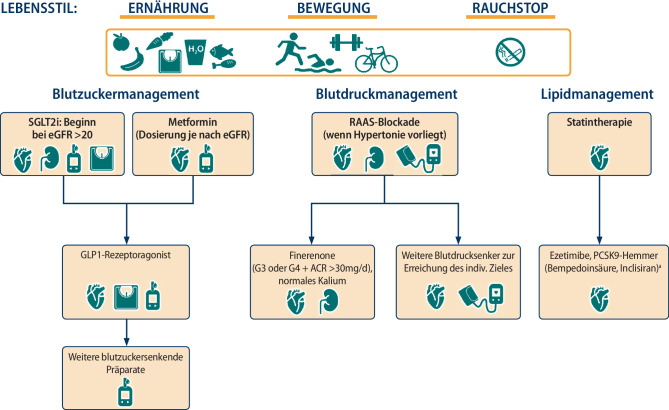

### Renoprotektive antihyperglykämische Substanzen

Einige antihyperglykämische Substanzen haben direkte renale Effekte gezeigt, die sich nicht alleine durch die Blutzuckersenkung erklären lassen [63].

#### SGLT-2-Inhibitoren

Empagliflozin, ein Vertreter der SGLT2-Inhibitoren, zeigte in der EMPA-REG-OUTCOME-Studie eine signifikante, 39 %ige relative Risikoreduktion im kombinierten Endpunkt bestehend aus Progression zu Makroalbuminurie, Verdopplung des Serum-Kreatinins, Beginn einer Nierenersatztherapie oder Tod renaler Ursache [64]. Canagliflozin reduzierte in der CANVAS Studie das Risiko für einen 40%igen eGFR-Abfall, den Beginn einer Nierenersatztherapie oder renalen Tod um relative 40 % [65]. Der nahezu idente Endpunkt (40%iger eGFR Abfall unter 60 ml/min/1,73 m^2^, Beginn einer Nierenersatztherapie und renaler Tod) wurde in der DECLARE-TIMI58 Studie mit Dapagliflozin um 47 % reduziert [66].

Nachdem obige Studien in Kollektiven von Menschen mit T2D durchgeführt wurden, untersuchten DAPA-CKD, CREDENCE und EMPA-KIDNEY die Effekte von Dapagliflozin, Canagliflozin und Empagliflozin bei Menschen mit CKD. In diesen Kollektiven konnte jeweils der primäre renale Endpunkt signifikant reduziert werden [67–69]. Während in der CREDENCE-Studie nur Personen mit T2D eingeschlossen wurden, hatten in DAPA-CKD etwa 68 % und EMPA-KIDNEY 46 % einen T2D.

Auch wenn die antihyperglykämischen Effekte bei SGLT-2-Inhibitoren mit abnehmender eGFR nachlassen, so bleiben die kardio-renoprotektiven Effekte bis zu einer eGFR von zumindest 20 ml/min/1,73 m^2^ erhalten.

#### GLP-1-Rezeptoragonisten

Von den Vertretern der GLP-1-Rezeptoragonisten hat Liraglutide in der LEADER-Studie den kombinierten renalen Endpunkt (persistierende Albuminurie > 300 mg/g, Verdopplung des Serum-Kreatinins, terminale Niereninsuffizienz oder Tod aufgrund terminaler Niereninsuffizienz) um relative 22 % im Vergleich zur Plazebogruppe (i.e. Blutzuckersenkung ohne GLP-1-RA) reduziert, ein Effekt, der in erster Linie durch eine Reduktion im Auftreten einer Makroalbuminurie getragen wird [70]. Semaglutide und Dulaglutide bestätigte diesen Effekt in der SUSTAIN-6-Studie bzw. der REWIND Studie [71, 72]. Ob ein tatsächlicher günstiger Einfluss dieser Substanzklasse auf die CKD Progression exisitiert wird in einer gerade laufenden Studie untersucht (FLOW trial).

### Therapiebesonderheiten bei nachlassender Nierenfunktion

Die Auswahl von oralen Antidiabetika hat in den letzten Jahren deutlich zugenommen. Dennoch gestaltet sich die orale antidiabetische Therapie bei Nierenfunktionseinschränkung schwieriger als bei diabetischen Patienten mit normaler Nierenfunktion [73]. Ebenso ist auf die erhöhte Hypoglykämieneigung in diesem Zusammenhang Aufmerksamkeit zu legen [74]. Im Folgenden werden die wesentlichen Substanzen bzw. Substanzgruppen aufgelistet:

- Metformin galt lange Zeit aufgrund seiner Plasmaeliminations-Halbwertszeit von 4,0–8,7 h [75] bei kompletter renaler Elimination bei mittel- bis höhergradiger Nierenfunktionseinschränkung aufgrund der Laktatazidosegefahr als kontraindiziert. Dies ändere sich allerdings über die letzten Jahre da dafür die Evidenz aus der klinischen Praxis fehlte [76, 77]. Metformin ist bei einer eGFR < 30 ml/min/1,73 m^2^ kontraindiziert, unter einer eGFR von 45 ml/min/1,73 m^2^ sollte Metformin nicht neu begonnen werden, die Dosis bei bestehender Therapie auf 1000 mg am Tag beschränkt und die eGFR engmaschiger überwacht werden. Studien zur Verwendung von Metformin bei eingeschränkter Nierenfunktion unterstützen diese Vorgangsweise [78] und empfehlen im Stadium 3b die Aufteilung der 1000-mg-Tagesmaximaldosis auf zweimal täglich 500 mg [79].
- SGLT-2-Inhibitoren (Indikation Blutzuckersenkung): Nachdem wie bereits erwähnt die blutzuckersenkende Wirkung dieser Substanzklasse mit eGFR-Verringerung abnimmt, sollte ab einer eGFR von < 45 ml/min/1,73 m^2^ wenig Erwartung in eine weitere blutzuckersenkende Potenz gesetzt werden. Eine SGLT-2-Inhibitor-Therapie sollte jedoch bei einer eGFR < 45/ml/min/1,73m^2^ fortgeführt werden, da die günstigen renalen Effekte zumindest bis zu einer eGFR von 20 ml/min/1,73m^2^ weiterhin erhalten bleiben. Eine Therapieeinleitung erscheint bei einer eGFR von > 20 ml/min/1,73m^2^ sinnvoll und die Fortführung einer bereits bestehenden Therapie ist bis zum Beginn einer Nierenersatztherapie möglich [37].
- Für GLP-1-Rezeptoragonisten gilt: Dulaglutide, Liraglutide und Semaglutide können bis zu einer eGFR von 15 ml/min/1,73m^2^ ohne Dosisanpassung angewendet werden, für terminale Niereninsuffizienz liegen bislang noch keine ausreichenden Daten vor. Für Lixisenatide ist bis zu einer eGFR von 30 ml/min/1,73m^2^ ebenfalls keine Dosisanpassung notwendig (aktuelle Fachinformationen). Exenatid 1 -mal wöchentlich sollte nach aktueller Datenlage bei Patient*innen mit einer eGFR < 30 ml/min/1,73m^2^ nicht eingesetzt werden.
- Für DPP-4-Hemmer gilt: Linagliptin kann in allen Stadien ohne Dosisanpassung gegeben werden, da es primär hepatobiliär ausgeschieden wird. Bei anderen DPP-4-Hemmern wie Sitagliptin, Vildagliptin, Saxagliptin und Alogliptin sind ab Stadium G3 Dosisanpassungen erforderlich.
  Sulfonylharnstoffe (SH) stellen aufgrund des Hypoglykämierisikos nicht das optimale orale Antidiabetikum bei Patient*innen mit CKD dar. Zwischen den einzelnen Substanzen gibt es erhebliche Unterschiede. Gliclazid sollte bei CKD in niedriger Dosierung begonnen und alle 4 Wochen dosistitriert werden. Glimepirid kann im Stadium CKD G1–3 in normaler Dosis, im Stadium G4 reduziert (1 mg/Tag) verabreicht werden und sollte im Stadium G5 vermieden werden [80]. Das Hypoglykämierisiko erscheint am niedrigsten bei Gliclazid [81], gefolgt von Glipizid und Glimepirid [82]. Dennoch ist insgesamt das Hypoglykämierisiko unter SH 10-fach so hoch wie unter Metformin und 4‑ bis 5‑fach höher als unter Pioglitazon [83–86].
  Es sollte auf die Gabe des vorwiegend renal eliminierten Glibenclamid verzichtet werden (heutzutage kaum mehr verwendet) wegen der Kumulationsgefahr mit Neigung zu schwerer und protrahierter Hypoglykämie.
- Bei Verwendung von Repaglinid kann bis CKD Stadium G4 ohne Dosisreduktion vorgegangen werden. Für Repaglinid gibt es im Stadium CKD G5 keine Daten.
- Pioglitazon als einzig verbleibender Vertreter der Thiazolidindione muss nicht dosisreduziert werden [80]. Pioglitazon kann entsprechend der Fachinformation bei einer Kreatinin-Clearance > 4 ml/min eingesetzt werden. Aufgrund des erhöhten Risikos für Herzinsuffizienz durch Volumenretention und dem erhöhten Risiko für periphere Frakturen, ist der Einsatz von Pioglitazon jdeoch limitiert.
- Bei Insulinen ist auf eine mögliche Dosisreduktion in Abhängigkeit von der Nierenfunktionseinschränkung zu achten, da Insulin teilweise renal abgebaut wird und das Hypoglykämierisiko bei weit fortgeschrittener CKD generell signifikant erhöht ist.

## Blutdruckeinstellung

Eine antihypertensive Behandlung von Diabetespatient*innen hat das Ziel, Auftreten und Progression einer DKD sowie makrovaskuläre Komplikationen und vorzeitigen kardiovaskulären Tod zu vermeiden. Daraus ergeben sich folgende Therapieziele: Rückbildung bzw. Stabilisierung einer Albuminurie; Erhalt der Nierenfunktion; Verhinderung der terminalen Niereninsuffizienz; Reduktion kardiovaskulärer Morbidität und Mortalität. Blutdrucktherapieempfehlungen für Kinder werden im pädiatrischen Kapitel erläutert.

Der Zielblutdruck bei diabetischer Nierenerkrankung wird mit < 140/90 mm Hg angegeben, um die kardiovaskuläre Mortalität und die Progression der CKD zu reduzieren. Zusätzlich wird von KDIGO bei einer Albuminurie > 30 mg/g ein Zielblutdruck von < 130/80 mm Hg vorgeschlagen [87, 88]. Eine Unterstützung für diese Zielwerte ergibt sich aus einer limitierten Anzahl von randomisierten Studien, welche auch Patienten mit Diabetes mellitus beinhalteten und sich auf kardiovaskuläre Ereignisse fokussierten [51]. Allerdings existieren keine randomisierten Studien hinsichtlich Zielblutdruckwerten, die auf renale Ereignisse eingehen. Daten, welche eine Progressionsverzögerung der CKD zeigen, stammen ausschließlich von drei randomisierten Studien bei Patient*innen ohne DKD, welche Afroamerikaner mit hypertensiver Nephropathie, Patienten mit IgA-Nephropathie und Patient*innen mit CKD ohne spezifische Diagnose umfassten [89].

Es gab auch ein Gefahrensignal aus klinischen Studien, dass diastolische Blutdruckwerte < 70 mm Hg und insbesondere < 60 mm Hg bei älteren Patient*innen problematisch sein könnten [90]. Daten von Patient*innen mit CKD Stadium G3 oder höher zeigten, dass ein diastolischer Blutdruckwert < 60 mm Hg mit einer erhöhten Inzidenzrate an terminaler Niereninsuffizienz vergesellschaftet ist [91], während andere Studien bei Patient*innen ohne CKD bei diastolischen Werten < 65 mm Hg eine Assoziation mit schlechterem Outcome der kardiovaskulären Erkrankungen zeigten [88, 92].

Ein therapeutischer Nutzen von Blockern des RAAS, sei es durch Verwendung eines ACE-Hemmers oder Angiotensin-Rezeptorblockers, ist durch eine Fülle von klinischen Daten nachgewiesen, insbesondere hinsichtlich der Reduktion von renalen Ereignissen bei Patienten im CKD Stadium G3 oder höher, solchen mit einer Albuminurie, Hypertonie und Diabetes mellitus [7, 8, 93]. Daher stellen diese die First-line-Therapie einer antihypertensiven Therapie dar, auch wenn es Hinweise gibt, dass andere Antihypertensiva gleichwertig hinsichtlich harter kardiovaskulärer Endpunkte und dem Auftreten von terminalem Nierenversagen wären [94]. Gegensätzlich zur aufgestellten Hypothese, dass eine RAAS-Doppelblockade klinisch sinnvoll wäre, mussten klinische Studien vorzeitig aufgrund von höheren Raten an Hyperkaliämie und/oder akutem Nierenversagen und fehlender Effizienz gestoppt werden [95–97].

## Mineralokortikoid-Rezeptor Antagonisten Therapie

Aktuell stehen zwei Klassen an Mineralokortikoid-Rezeptor Antagonisten zur Verfügung – die steroidalen und die nicht-steroidalen. Rezent hat die FIDELIO-DKD Studie die renalen Effekte von Finerenone bei Personen mit diabetischer Nierenerkrankung untersucht. Der primäre Endpunkt (Nierenversagen, zumindest 4 Wochen anhaltender Abfall der eGFR um > 40 %, renaler Tod) wurde signifikant um relative 18 % reduziert [98]. Diese renalen Ergebnisse wurden von einer zweiten kardiovaskulären Outcome-Studie (FIGARO) bestätigt. Der kombinierte, primäre kardiovaskuläre Endpunkt wurde in dieser Studie um signifikante relative 24 % gesenkt [99]. Das Risiko für akutes Nierenversagen unterschied sich nicht signifikant zwischen der Finerenone- und der Plazebogruppe und die Häufigkeit von Hyperkaliämien, die zu einem Studienabbruch führten, war 0,6 % unter Placebo und 1,7 % in der Finerenone-Gruppe in beiden Studien zusammengefasst [100].

Eine Finerenone-Therapie kann bei Menschen mit Diabetes mellitus und CKD G3–4 eingesetzt werden, die trotz einer Therapie mit einem ACE-Hemmer oder Angiotensin-Rezeptorblocker für zumindest vier Wochen eine persistierende Albuminurie (A2–3) und normale Kaliumwerte aufweisen. Über einen möglichen synergistischen Effekt von SGLT2-Inhibitoren und Finerenone liegen noch keine aussagekräftigen Daten vor, da nur wenige Patient*innen mit dieser Kombination in den Studien behandelt wurden.

## Zusammenfassung

Zielwerte und Maßnahmen bei diabetischer Nierenerkrankung:

### Blutdruck

- RR < 140/90 mm Hg
- RR < 130/80 mm Hg bei Albuminurie (Stadium A2 und A3)
- RR diastolisch > 60 mm Hg

### HbA1c-Zielwerte

- HbA_1c_-„Zielkorridor“ meistens 6,5–7,5 % (48–58 mmol/mol) (bei fortgeschrittener CKD)
- HbA_1c_-„Zielkorridor“ bei Dialysepatient*innen 7–8,0 % (53–64 mmol/mol). Dieser soll entsprechend dem Alter und der Komorbiditäten individualisiert werden.

### LDL-Cholesterin-Ziel

- Bei Diabetes mellitus mit Albuminurie, CKD G3 oder G4: < 55 mg/dl

### Weitere Aspekte

- Hämoglobin 9–11 g/dl (eGFR Stadium CKDG 4–5)
- Elektrolyte im Normbereich
- Normalisierung der Eiweißzufuhr auf täglich 0,8–1,3 g/kg Körpergewicht
- Thrombozytenaggregationshemmer (individuelle Abwägung des potenziellen kardiovaskulären Benefits gegenüber dem Blutungsrisiko)
- Verzicht auf Rauchen
- Exakte Nutzen-Risiko-Abwägung vor Einsatz potenziell nephrotoxischer Medikamente (z. B. nichtsteroidale Antirheumatika, bestimmte Antibiotika)
- Protektive Maßnahmen bei Röntgenkontrastmittelgabe wegen der erhöhten Gefahr einer akuten Nierenschädigung (CT mit KM: bei eGFR < 30 ml/min/1,73^2^; bei arteriellen Angiographien eGFR < 45 ml/min/1,73 m^2^): auf ausreichende Hydrierung achten
- Beachten der möglichen Kumulation von Begleitmedikamenten
- Beachten des erhöhten kardiovaskulären Risikos mit Screening für Angiopathie
- Beachten von Harnwegsinfekten (Restharn?)

### Kontrollen bei Patienten mit diabetischer Nierenerkrankung

Je nach CKD-Stadium und Progression mindestens 2‑ bis 4 -mal jährliche Kontrollen:

- HbA_1c_, Lipide
- Bestimmung der Albuminurie bzw. Albumin-Kreatinin-Ratio
- Bestimmung der Retentionsparameter und Serumelektrolyte (Kreatinin, Harnstoff oder BUN, Kalium)
- Bestimmung der eGFR
- Blutdruckselbstmessung mit Protokollierung, empfohlen ambulante 24-h-Blutdruckmessung

Bei einer eGFR < 60 ml/min/1,73 m^2^ zusätzlich (Frequenz vom CKD Stadium abhängig):

- Blutbild
- Eisenstatus mit Ferritin, Transferrin, Transferrinsättigung, Serumeisen
- Serum-Phosphat, Serum-Kalzium
- Parathormon, 25-OH Vitamin D
- Bestimmung der venösen Blutgase insbesondere bei eGFR < 30 ml/min
- Serumkalium (vor allem beim Einsatz von RAAS-blockierenden Antihypertensiva und auch Mineralokortikoid-Rezeptor Antagonisten)
- Interdisziplinäre diabetologisch-nephrologische Betreuung ab eGFR < 60 ml/min (Stadium G3) erwägen (Details siehe Text oben)
- Hepatitis-B-Virus-Impfschutz
- Bei Auftreten einer akuten Niereninsuffizienz bzw. Verdacht auf das Vorliegen einer nicht-diabetischen Nierenerkrankung (signifikante Proteinurie) ist eine umgehende nephrologische Begutachtung des Patienten zu veranlassen
- Zur Diagnosesicherung und optimalen Therapieempfehlung ist oftmals eine Nierenbiopsie indiziert. Dieses Vorgehen wird im Einzelfall vom Nephrologen mit dem Patienten besprochen.

## Interessenkonflikt

H. Sourij hat von folgenden Unternehmen, die auch tlw. fördernde Mitglieder der ÖDG sind, Forschungsunterstützungen und/oder Honorare für Vorträge oder Beratertätigkeit erhalten: Amarin, Amgen, AstraZeneca, Boehringer-Ingelheim, Bayer, Daiichi Sankyo, Eli Lilly, Kapsch, Merck Sharp & Dohme, Novo Nordisk, Sanofi-Aventis. R. Edlinger hat von folgenden Unternehmen Kongreßunterstützung, Honorar für Vorträge oder Beratertätigkeit erhalten: Amgen, Astellas, AstraZeneca, Boehringer-Ingelheim, Fresenius-Kabi, Novartis, Sanofi-Aventis, Vifor. F.C. Prischl hat von folgenden Unternehmen Reiseunterstützungen oder Honorar für Vorträge oder Advisory Boards erhalten: Amgen, Astellas, AstraZeneca, Boehringer-Ingelheim, Bayer Pharma, Eli Lilly, Fresenius Medical Care, Gambro, Janssen-Cilag Pharma, Novartis, ratiopharm, Roche, Takeda Pharma, Wyeth-Lederle. S. Kaser hat von folgenden Unternehmen, die zT auch fördernde Mitglieder der ÖDG sind, Forschungsunterstützungen und/oder Honorare erhalten: Amarin, AstraZeneca, Boehringer Ingelheim, Eli Lilly, Merck Sharp & Dohme, Novartis, Novo Nordisk, Viatris. S. Horn hat von folgenden Unternehmen Reiseunterstützung, Honorare für Vorträge oder Beratertätigkeit erhalten: Astra Zeneca, Amgen, Bayer Pharma, Boehringer Ingelheim, Daiichi Sankyo, Fresenius, GlaxoSmithKline, Janssen Cilag, Novartis, Novo Nordisk, Otsuka, Pfizer, Vifor. M. Antlanger hat von folgenden Unternehmen Reiseunterstützung, Honorare für Vorträge oder Beratertätigkeit erhalten: Astra Zeneca, Amgen, Baxter, Boehringer Ingelheim, GlaxoSmithKline, Janssen Cilag, Novartis, Vifor. B. Paulweber hat von folgenden Unternehmen Honorare für Vorträge oder Beratertätigkeit erhalten: Amgen, AstraZeneca, Boehringer-Ingelheim, Eli Lilly, Merck Sharp Dohme, Mylan, Novartis, Novo Nordisk, Pfizer, Sanofi-Aventis. F. Aberer hat von folgenden Unternehmen Reiseunterstützung, Honorare für Vorträge oder Beratertätigkeit oder Forschungsunterstützungen erhalten: Amgen, Sanofi, Novo Nordisk, Astra Zeneca und Boehringer Ingelheim. H. Stingl hat von folgenden Unternehmen, die teils auch fördernde Mitglieder der ÖDG sind, Forschungsunterstützungen, Honorare für Vorträge und/oder Beratertätigkeit, bzw. Fortbildungsunterstützung im Rahmen der (dienst)rechtlichen Rahmenbedingungen (Einladung zu Kongressen) erhalten: Abbott, Amgen, AstraZeneca, Boehringer Ingelheim, Daiichi Sankyo, Eli Lilly, Menarini, MSD, Mylan, Novo Nordisk, Novartis, Sanofi Aventis, Servier. Zusätzlich gibt H. Stingl an, dass für ihn persönlich kein Interessenkonflikt vorliegt. A. Kautzky-Willer hat von folgenden Unternehmen, die teils auch fördernde Mitglieder der ÖDG sind, Honorare für Vorträge und/oder Beratertätigkeit erhalten: Astra Zeneca, Böhringer-Ingelheim, Novo Nordisk, Lilly, Amgen, Novartis. S. Schmaldienst hat von folgenden Unternehmen, die teils auch fördernde Mitglieder der ÖDG sind, Honorare für Vorträge und/oder Beratertätigkeit erhalten: AstraZeneca und Böhringer-Ingelheim. M. Säemann hat von folgenden Unternehmen, die teils auch fördernde Mitglieder der ÖDG sind, Honorare für Vorträge und/oder Beratertätigkeit und Studienunterstützung erhalten: Astellas, AstraZeneca, Bayer, Boehringer-Ingelheim, Lilly, Novo Nordisk, Novartis, and Otsuka. M. Clodi hat von folgenden Unternehmen, die teils auch fördernde Mitglieder der ÖDG sind, Honorare für Vorträge und/oder Beratertätigkeit erhalten:Astra Zeneca, Boehringer Ingelheim, Eli Lilly, Sanofi, Novo Nordisk, Daiichi, Amgen, Abbott, Dexcom, Medtrust. G. Mayer hat von folgenden Unternehmen, die teils auch fördernde Mitglieder der ÖDG sind, Honorare für Vorträge und Forschungsunterstützung erhalten: AstraZeneca, Böhringer Ingelheim und NovoNordisk. R. Oberbauer, hat von folgenden Unternehmen, die teils auch fördernde Mitglieder der ÖDG sind, Honorare für Vorträge und/oder Beratertätigkeit und Studienunterstützung erhalten: AstraZeneca, Böhringer Ingelheim, Eli Lilly, Astellas. J. Brix, D. Cejka, und A. Rosenkranz geben an, dass kein Interessenkonflikt besteht.
